# Revisions of *Ruizodendron* and *Pseudephedranthus* (Annonaceae) including a new species and an overview of most up-to-date revisions of Neotropical Annonaceae genera

**DOI:** 10.3897/phytokeys.86.13773

**Published:** 2017-09-21

**Authors:** Roy H.J. Erkens, Jessica Oosterhof, Lubbert Y.T. Westra, Paul J.M. Maas

**Affiliations:** 1 Maastricht Science Programme, Maastricht University, Maastricht, The Netherlands; 2 Unaffiliated, The Netherlands; 3 Naturalis Biodiversity Center, Leiden, The Netherlands

**Keywords:** Biodiversity, Neotropics, new species, systematics, taxonomy

## Abstract

We present revisions of the Neotropical genera *Ruizodendron* and *Pseudephedranthus* (Annonaceae). *Ruizodendron* includes a single species *R. ovale*. *Pseudephedranthus* now comprises two species, with the description of the new species *P. enigmaticus* **sp. nov.** extending the range of the genus beyond the Upper Rio Negro region of Brazil (Amazonas) and adjacent Venezuela (*P. fragrans*), to include Guyana, Suriname, and the Brazilian state of Pará. An overview is provided of current revisions of Neotropical Annonaceae genera that will aid in accessing proper species information for this frequently encountered tropical rain forest family.

## Introduction

Annonaceae are frequent components of tropical rain forests worldwide (Gentry 1993; Tchouto et al. 2006; Punyasena et al. 2008; Sonké and Couvreur 2014), for which it is vital to have a good overview of the most recent knowledge of the ca. 2450 species (Rainer and Chatrou 2006). Revisions and monographs are an important tool to document this knowledge, be it online or in a more traditional printed format. This documentation is not a trivial exercise. For instance, Ter Steege et al. (2016) wanted to estimate how many tree species occur in the Amazon basin. They used, amongst many other sources, the most recent revision of the Annonaceae genus *Guatteria* (Maas et al. 2015) to check their preliminary taxa list. Since Maas et al. (2015) reduced 62 Amazonian tree species into synonymy and described 10 new Amazonian species the check of Ter Steege et al. reduced the taxonomic error for this group substantially.

With respect to Neotropical Annonaceae, renewed revisionary efforts have been underway since the early eighties (Maas 1984; Chatrou 1999; Oliveira and Sales 1999). To date, 29 out of 34 Neotropical genera (Maas et al. 2011) have been revised, of which 11 published after 2000 (Table 1). Revisionary work on three further genera (*Desmopsis* Saff., *Ephedranthus* S.Moore, and *Sapranthus* Seem.) is at an advanced stage, and the final two, *Annona* L. (including *Rollinia* A.St.-Hil.) and *Xylopia* L., are subject of ongoing research. The *Duckeanthus* R.E.Fr. revision (Fries 1934) is the oldest still in use and together with a more recent addition by Maas et al. (1993) provides an adequate summary of all knowledge available on this monospecific genus. However, not all recently revised genera might be monophyletic (e.g. *Desmopsis*, *Klarobelia* Chatrou, *Oxandra* A.Rich. and *Stenanona* Standl.) and some of the taxonomic treatments will need to be modified in the light of future molecular phylogenetic evidence.

Two genera so far still awaiting revision were the monospecific genus *Ruizodendron* R.E.Fr. and the non-related genus *Pseudephedranthus* Aristeg.

**Table 1.** 34 Neotropical genera of Annonaceae (based on Maas et al. 2011) and their most up-to-date revision. A more elaborate overview of taxonomic literature of these taxa can be found in Erkens et al. (2012).

**Genus**:	**Most up-to-date revision**:
*Anaxagorea* A.St.-Hil.	Maas and Westra 1984, 1985 with a new species in Maas et al. 1986
*Annona* L. (including *Rollinia* A.St.-Hil.)	Ongoing work (for inclusion of *Rollinia* see Rainer 2007)
*Asimina* Adanson (including *Deeringothamnus* Small)	Kral 1960 (for inclusion of *Deeringothamnus* see Maas et al. 2011)
*Bocagea* A.St.-Hil.	Johnson and Murray 1995
*Bocageopsis* R.E.Fr.	Maas et al. 2007
*Cardiopetalum* Schltdl.	Johnson and Murray 1995
*Cremastosperma* R.E.Fr.	Pirie et al. 2005
*Cymbopetalum* Benth.	Murray 1993
*Desmopsis* Saff.	Schatz et al. in prep. (pers. comm.)
*Diclinanona* Diels	Erkens et al. 2014
*Duckeanthus* R.E.Fr.	Fries 1934; Maas et al. 1993
*Duguetia* A.St.-Hil.	Maas et al. 2003, including all African species with a new species in Maas and Westra 2010, and a new species in Bazante and Alves 2017
*Ephedranthus* S.Moore	Carvalho Lopes in prep. (pers. comm.)
*Froesiodendron* R.E.Fr.	Johnson and Murray 1995
*Fusaea* (Baill.) Saff.	Chatrou and He 1999
*Guatteria* Ruiz & Pav.	Maas et al. 2015
*Hornschuchia* Nees	Johnson and Murray 1995
*Klarobelia* Chatrou	Chatrou 1998
*Malmea* R.E.Fr.	Chatrou 1998
*Mosannona* Chatrou	Chatrou 1998
*Onychopetalum* R.E.Fr.	Maas et al. 2007
*Oxandra* A.Rich.	Junikka et al. 2016
*Porcelia* Ruiz & Pav.	Murray 1993
*Pseudephedranthus* Aristeg.	Oliveira and Sales 1999 and this work
*Pseudomalmea* Chatrou	Chatrou 1998
*Pseudoxandra* R.E.Fr.	Maas and Westra 2003 with a new species in Maas and Westra 2005
*Ruizodendron* R.E.Fr.	This work
*Sapranthus* Seem.	Schatz et al. in prep. (pers. comm.)
*Stenanona* Standl.	Schatz and Maas 2010 with a new species in Ortiz-Rodriguez et al. 2014
*Tetrameranthus* R.E.Fr.	Westra and Maas 2012
*Tridimeris* Baill.	Schatz 1987 with a new species in Ortiz-Rodriguez et al. 2016
*Trigynaea* Schltdl.	Johnson and Murray 1995 with a new species in Lobão 2017
*Unonopsis* R.E.Fr.	Maas et al. 2007
*Xylopia* L.	Ongoing work

### Taxonomic history of *Ruizodendron*

**Figure 1.**
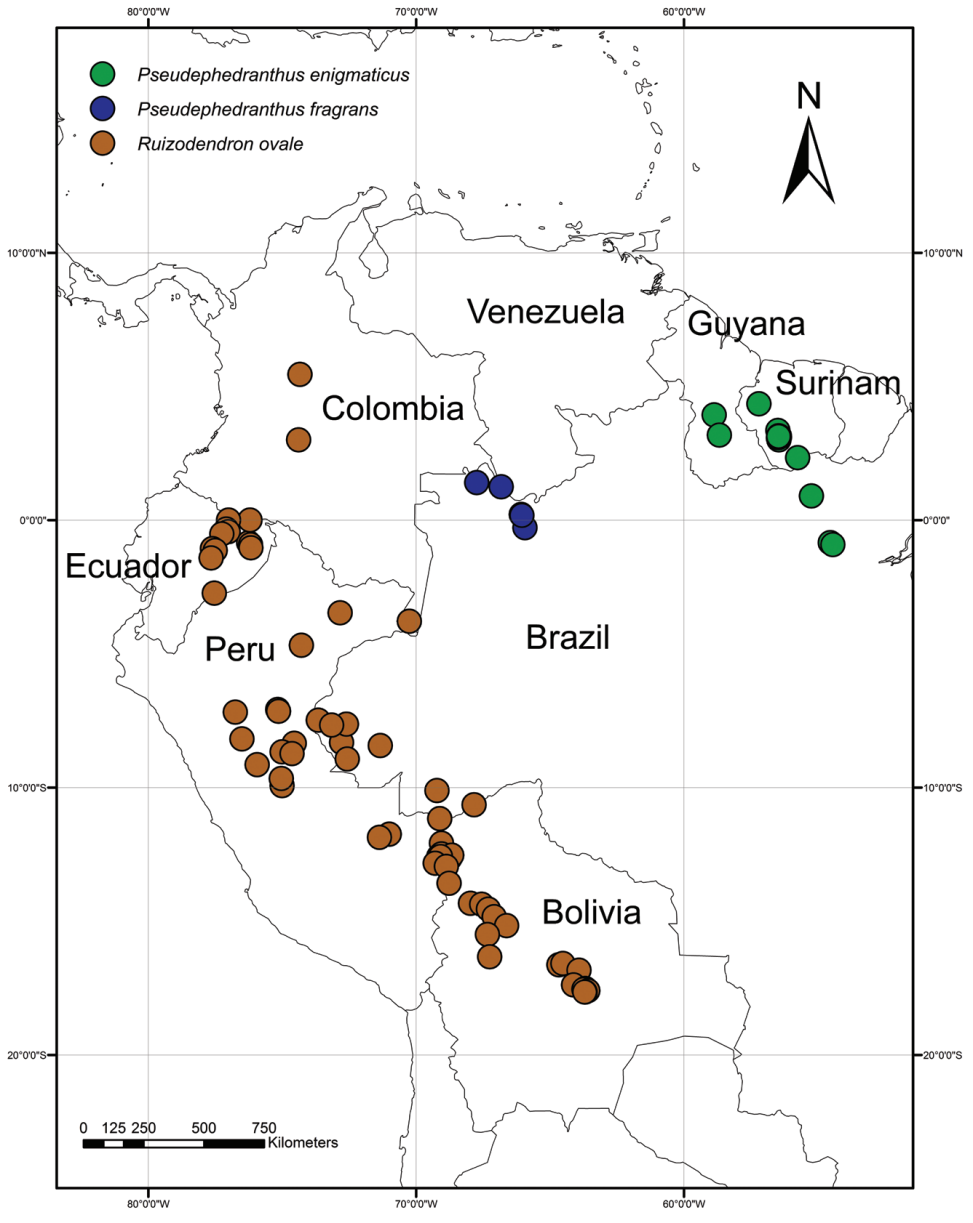
Distribution map of *Ruizodendron ovale*, *Pseudephedranthus fragrans* and *P. enigmaticus*.

**Figure 2.**
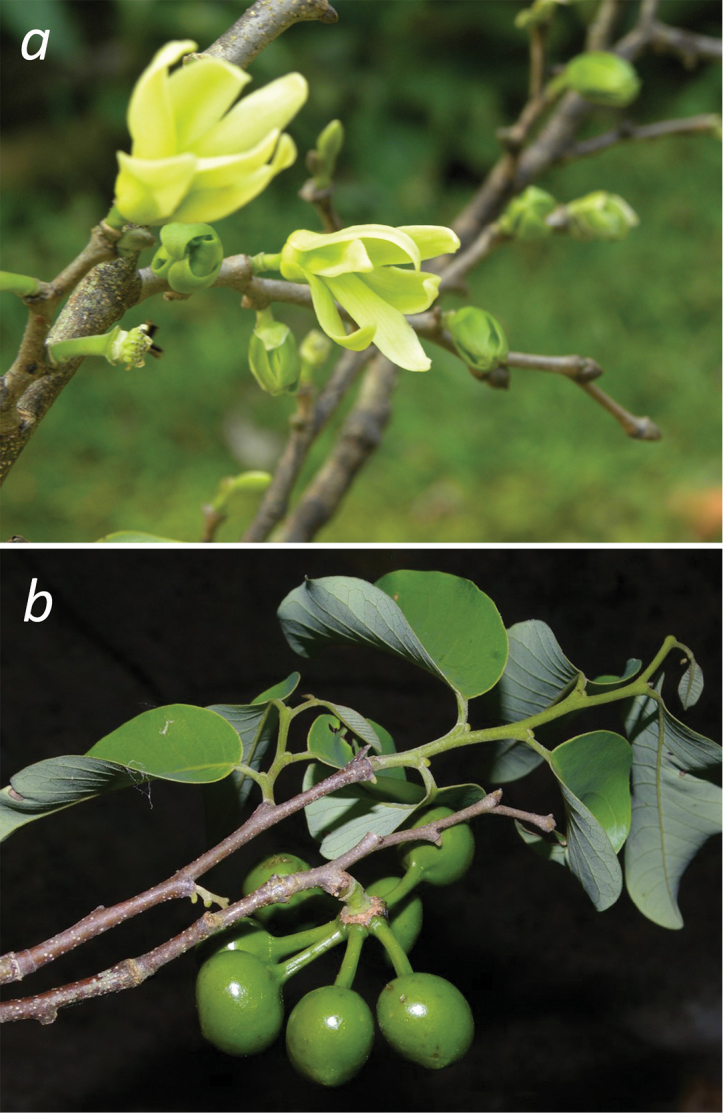
**a** Flowering branch of *Ruizodendron ovale* **b** The asymmetrical, transversely-ellipsoid monocarps of *Ruizodendron ovale* (*van der Werff 22749*, MO). Missouri Botanical Garden, used with permission. Photograph 1b downloaded from http://www.tropicos.org. Photograph 1a taken by Abel Monteagudo, 1b taken by Rodolfo Vásquez.

**Figure 3.**
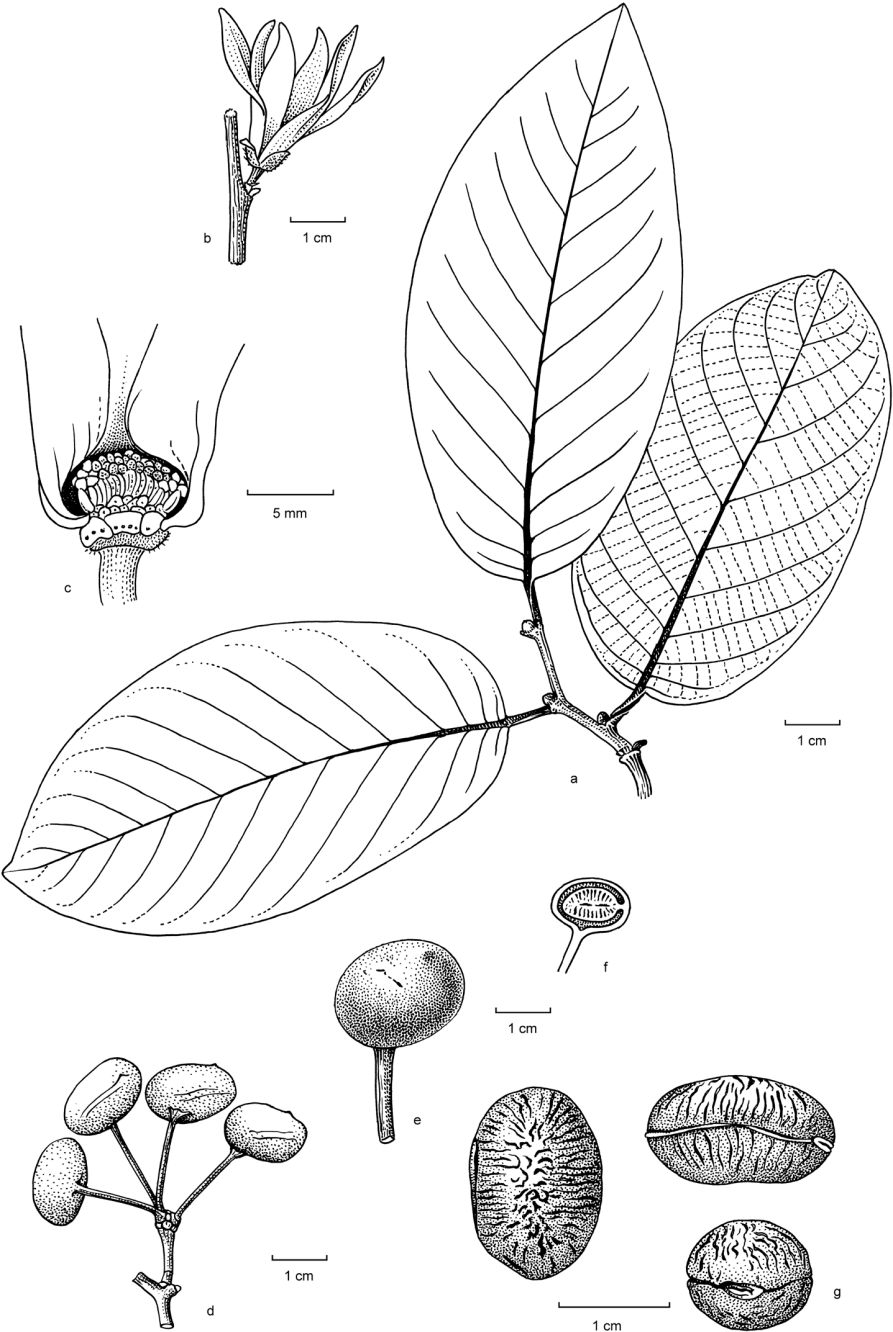
Drawing of *Ruizodendron ovale*. **a** Top of a branch **b** Flower **c** Base of flower with part of petals removed to show the interior **d** Fruit **e** Monocarp **f** Monocarp in longitudinal section showing laterally attached seed **g** Seed viewed clockwise starting from right: longitudinal view, hilum side, and in longitudinal section. Drawings by H. Rypkema.

The monospecific genus *Ruizodendron* occurs from Amazonian Brazil to Bolivia, Peru, Ecuador, and Colombia (Fig. 1). It is readily recognized by the asymmetrical transversely ellipsoid monocarps (Fig. 2b, Fig. 3d, e, f). The genus name consists of the Greek word for tree (*dendron*) and part of the name of the Spanish botanist Hipolito Ruiz López (1754–1815). Ruiz undertook a field trip throughout South America from 1779 to 1788 together with J. Pavón after which they published Florae Peruvianae et Chilensis Prodromus (1794, reprinted 1797) and Flora Peruviana et Chilensis (1798).

*Ruizodendron* was not originally described as a separate genus. During the field expedition of Ruiz and Pavón in South America in 1778–1788 they collected a specimen near Pozuzo in Peru that they described as *Guatteria ovalis* Ruiz & Pav. (Fries 1936; Maas et al. 2011; Maas et al. 2015). The specimen bore solely fruits and the description was therefore incomplete: “G. foliis oblongis ovalibusque. Arbor quadriorgyalis.” The epithet “ovalis” was appropriate for their material since the type has oval, on both sides rounded, leaves (Fig. 3a) of ca. 10 cm long (Fries 1939, p. 544; although we now know that leaves can be found that are less typically oval). Fries studied this type specimen (B) and found that vegetative parts and structure of the monocarps differed from that typical of *Guatteria*, concluding that this specimen should not be attributed to that genus. On the basis of the axillary flower buds with imbricate petals, and stamen and carpel morphology he concluded that this species should be placed close to *Cremastosperma* R.E.Fr. (Fries 1936, 1939).

In 1925 the same species was collected by Steinbach in Santa Cruz in Bolivia, but also this specimen solely bore fruits. Only with the finding by Klug of a flowering specimen in the Upper (“oberen”) Río Huallaga region (San Martín; not far from the type locality of *Guatteria ovalis*) the placement of the genus within Annonaceae could be studied for the first time. From the flower it was clear that this indeed was not *Guatteria*. Fries (1936) even thought that the flowers were quite aberrant for Neotropical Annonaceae: they had very thin, long and narrow petals (“linear lanzettlich”; Fig. 2a, Fig. 3b). Based on the position of the articulation on the pedicel and the position of the bracts Fries (1939) stated that *Ruizodendron* was related to *Cremastosperma*. However, the fruiting pedicel was placed in the middle of the long side of the monocarp (“…indem der Same bei *Guatteria ovalis* quer gestellt und in horizontaler Fläche ausgestreckt zu sitzen kommt mit dem Stiel des Monokarpiums mitten an der Längseite befestigt”; Fig. 2b, 3d, f). Based on this fruit type Fries concluded that *Ruizodendron* was unique among American Annonaceae (“Wir erhalten dardurch ein Früchttyp, der allein dasteht unter den amerikanischen Annonaceen”). Furthermore, based on dried material he judged that the specimens had fleshy fruit *in vivo*. This was not similar to his dried material of *Cremastosperma* species, which had a dry, fragile pericarp. All this led Fries to describe *Ruizodendron ovale* (Ruiz & Pav.) R.E.Fr.

More recently, Van Setten and Koek-Noorman (1992) used fruit and seed features to place *Ruizodendron* as part of a group with six other Neotropical genera (be it together with other non-Neotropical genera): *Cremastosperma*, *Ephedranthus*, *Malmea* R.E.Fr., *Oxandra*, *Pseudephedranthus and Pseudoxandra* R.E.Fr. This is in line with an earlier grouping of Walker (1971) based on pollen characters and a concurrent conclusion by Van Heusden (1992) based on flower morphology. One year later, Keßler (1993) included *Ruizodendon* with *Oxandra*, *Pseudoxandra*, *Cremastosperma*, and *Ephedranthus* in his “*Oxandra* group”, characterised by axillary flowers, imbricate sepals and petals, sulcate pollen grains, a single basal or lateral ovule, and free monocarps.

At this moment, based on molecular phylogenetic work (Chatrou et al. 2012), *Ruizodendron* is placed in a well-supported clade within tribe Malmeeae Chatrou & R.M.K.Saunders (subfamily Malmeoideae Chatrou, Pirie, Erkens & Couvreur) with *Ephedranthus*, *Klarobelia*, *Mosannona*, *Oxandra*, *Pseudephedranthus*, and *Pseudomalmea* Chatrou, although its exact phylogenetic placement within this clade is not yet known.

### Taxonomic history of *Pseudephedranthus*

Until now, *Pseudephedranthus* consisted of a single species: *P. fragrans* (R.E.Fr.) Aristeg. known only from Brazil in the state of Amazonas (Upper Rio Negro Region, Rio Cauaburí), and adjacent Venezuela (Piedra de Cucuy; Fig. 1). In 1993, Maas collected a specimen (*Maas et al. 6878*, Fig. 6, 7) of *P. fragrans* during a botanical expedition in the Upper Rio Negro Region of Brazil and adjacent Venezuela and reported that the species was fairly common in the forested hills at the base of Piedra de Cucuy. Newly described here, *P. enigmaticus* Maas & Westra is distributed across Guyana, Suriname, and the Brazilian state of Pará (Fig. 1).

**Figure 4.**
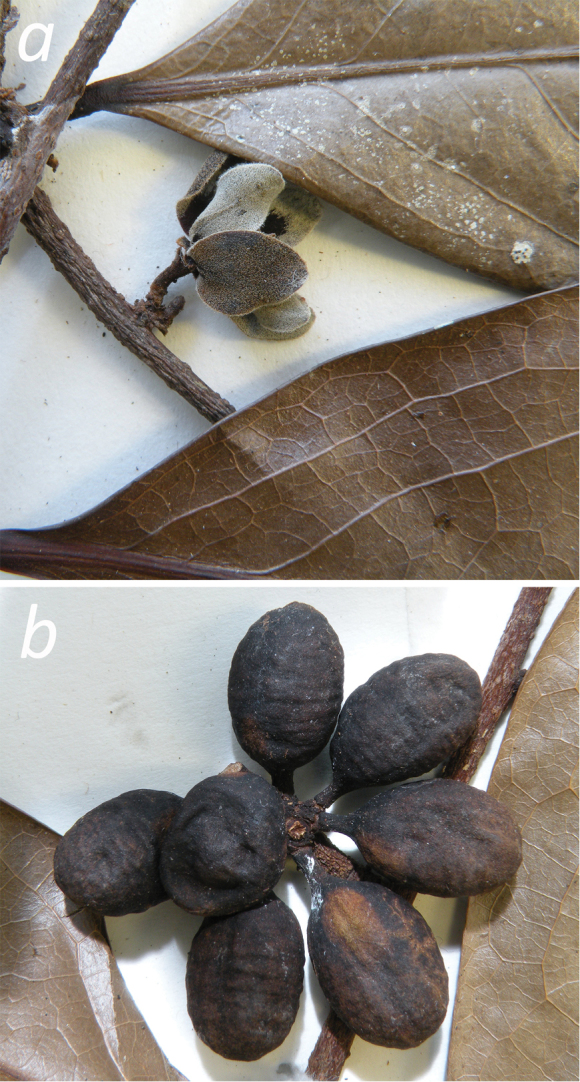
**a** Close up of flowering branch of *Pseudephedranthus enigmaticus* (*Evans et al. 3437*) **b** Close up of fruiting branch of *Pseudephedranthus enigmaticus* (*Herrera C. et al. 9959*).

*Pseudephedranthus* (Aristeguieta 1969) can be easily recognised vegetatively. Its leaves exhibit some quite distinctive features (Fig. 4a): 1) the lamina is shiny on the upper side; 2) the primary vein is both raised and ribbed at the upper side of the lamina; 3) relatively few, widely spaced secondary veins (5–9) are present, and 4) the upper side shows a reticulate venation. The latest revision dated from 1999 (Oliveira and Sales 1999), written in Portuguese.

**Figure 5.**
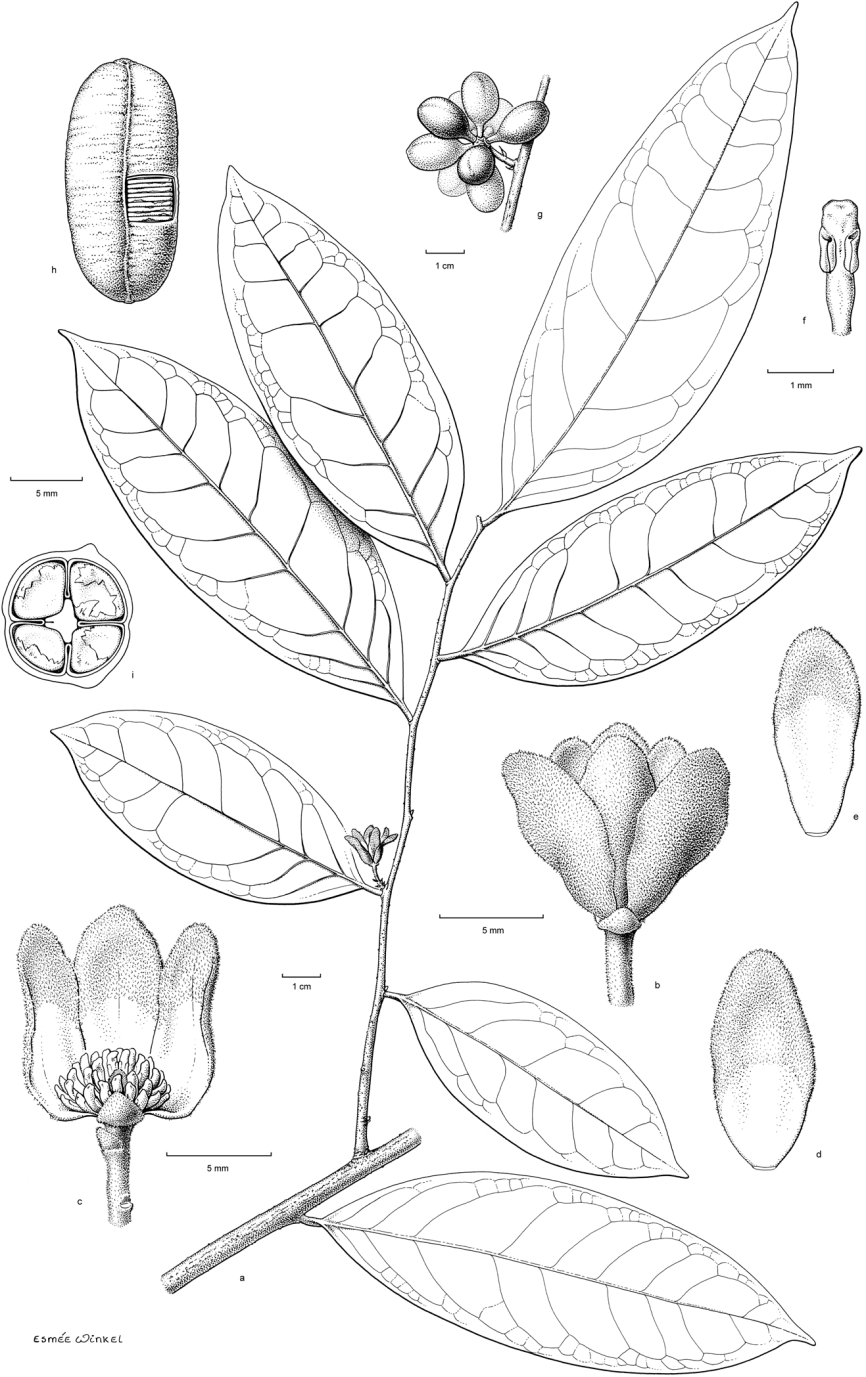
Drawing of *Pseudephedranthus enigmaticus*. **a** Flowering branch (*Clarke 3420*, U) **b** Flower in lateral view **c** Staminate flower in longitudinal section **d** Outer petal **e** Inner petal **f** Stamen (**b–f** *Evans et al. 3437*, WAG) **g** Fruit (*Herrera C. 9959*, WAG) **h** Seed, small part of seed coat removed to show lamellate ruminations **i** Cross section of monocarp and enclosed seed showing 4-parted rumination (**h–i** *Strudwick et al. 3808*, U). Drawings by Esmée Winkel.

Maas et al. (1993) hypothesised that *Pseudephedranthus* is closest to *Oxandra* by its elongate connective appendages, and to *Ephedranthus* by its androdioecious flowers and its extremely large seeds (25–30 × 13 mm, Fig. 5b).

Pirie et al. (2006) already showed that *Pseudephedranthus* is nested within *Klarobelia*. More recent molecular phylogenetic work (Chatrou et al. 2012) confirmed this and demonstrated as well that *Pseudephedranthus* is part of the same clade as *Ruizodendron* together with *Ephedranthus*, *Klarobelia*, *Mosannona*, *Oxandra*, and *Pseudomalmea* (in tribe Malmeeae Chatrou & R.M.K.Saunders).

## Materials and methods

Herbarium material was investigated from the following herbaria: A, AAU, CGE, E, F, G, GB, GH, INPA, K, L, LPB, MICH, MO, P, NY, RB, S, U, UC, US, WAG, and WIS. Dried herbarium material was used for measurements, colour indications and descriptions of surface structures. If measurements were done on material kept in spirit this is indicated by curly brackets { }. We have indicated the density of hair cover according to the following gradations: densely, rather densely and sparsely.

## Taxonomic treatment

### Ruizodendron

Taxon classificationPlantaeMagnolialesAnnonaceae

R.E.Fr.

Ruizodendron R.E.Fr., Arkiv för Botanik Ser. 28B (4): 3. 1936.

#### Type.

*Ruizodendron ovale* (Ruiz & Pav.) R.E.Fr. (= *Guatteria ovalis* Ruiz & Pav.)

#### Description.

*Trees*, often with buttresses; young twigs lenticellate. *Leaves*: distichous, simple, entire, shortly petiolate, exstipulate; lamina medium-sized, mostly elliptic, venation brochidodromous to eucamptodromous, articulated at the base, primary vein slightly raised to flat above, glaucous below. *Flowers* actinomorphic, bisexual, perianth consisting of one whorl of sepals and two whorls of petals; pedicels with basal articulation and 2 soon falling bracts; sepals 3, imbricate, free, much shorter than the petals, soon falling; petals 6, imbricate, free, subequal; stamens ca. 40, extrorse, filament very short, connective apex discoid, glabrous; carpels ca. 25, spirally arranged, free, ovary 1-locular with 1 ovule attached below the middle, style absent, stigma ovoid, hairy. *Fruit* apocarpous, composed of few indehiscent monocarps, these transversely ellipsoid, the stipe laterally attached. *Seed* 1, filling the whole monocarp, orange-brown, rumination lamellate in 2 parts, raphe a fine, shallow groove.

#### Distribution.

Monotypic, occurring in the western part of South America (Fig. 1).

### Ruizodendron ovale

Taxon classificationPlantaeMagnolialesAnnonaceae

(Ruiz & Pav.) R.E.Fr.

Figs 2 , 3

Ruizodendron ovale (Ruiz & Pav.) R.E.Fr., Arkiv för Botanik Ser. 28B (4): 3. 1936. – Guatteria ovalis Ruiz & Pav., Systema vegetabilium florae peruvianae et chilensis 1: 146. 1798.

#### Type.

PERU. Huánuco: Pozuzo, anno 1782, *Ruiz & Pavón s.n.* (holotype MA [barcode MA811639], only photograph seen; isotype B).

#### Description.

*Tree*, 5–45 m tall, 10–70 cm diam., buttresses to ca. 1.5 m high, young twigs longitudinally fissured, sparsely covered with appressed, silvery hairs 0.1–0.4 mm long, soon glabrous. *Leaves*: petioles 5–10(–15) by 1–2 mm; lamina elliptic to ovate, rarely narrowly so, 6–25 by 4–12 cm (index 1.5–2(–3)), chartaceous, olive green to greenish brown and shiny above *in sicco*, glaucous to pale brown below, glabrous above, sparsely covered with appressed, silvery hairs to ca. 0.4 mm long mainly on larger veins below, base rounded with the extreme base slightly attenuate; apex obtuse, rarely acute; secondary veins 7–14 on either side of primary vein, slightly raised to flat above, shortest distance between secondary veins and margin 1–3 mm, tertiary veins strongly raised above, percurrent to reticulate. *Inflorescences* axillary, 1-flowered, rarely 2 together through auxiliary bud formation, often appearing on small, leafless lateral branches. Indument: pedicels, outer side of bracts and sepals densely covered with appressed, silvery hairs to ca. 0.4 mm long, petals sparsely so; pedicels 4–8 by 1 mm, fruiting pedicels to ca. 10 by 1–3 mm; bracts 2, soon falling, one bract placed just below the articulation, ovate, ca. 4 mm long, other bract placed at ca. 0.2 from the base of the pedicel, ovate-triangular, ca. 4 mm long; sepals ovate-triangular, 5–6 by 3–4 mm, soon falling; petals white to cream *in vivo*, black *in sicco*, narrowly elliptic, 20–50 by 3–5 mm, membranous, base widened enclosing the pollination chamber; torus slightly raised; stamens ca. 1.5 mm long; carpels ca. 1 mm long. *Monocarps* 1–10, green, maturing black *in vivo*, brownish black *in sicco*, transversely ellipsoid, 15–20{–25} by 10–16 mm, glabrous, apex excentrically apiculate (apiculum to ca. 1 mm long), wall 0.2–0.5 mm thick; stipes 8–25 by 1–1.5 mm, excentrically placed. *Seed* transversely ellipsoid, 14–22 by 9–13 mm, orange-brown.

#### Distribution.

Colombia (Amazonas, Cundinamarca, Meta), Ecuador (Morona-Santiago, Napo, Orellana, Pastaza, Sucumbios), Peru (Huánuco, Loreto, Madre de Dios, Pasco, San Martín, Ucayali), Bolivia (Beni, Cochabamba (?), La Paz, Pando, Santa Cruz), Brazil (Acre). Fig. 1.

#### Habitat and ecology.

In tropical rain forest, often on alluvial soil. At elevations of up to 800 m. Flowering: July to September; fruiting: August to May.

#### Vernacular names.

Bolivia: Blanquillo (*Vargas C. et al. 1133*), Fariña seca de bajío (*Dominguez & Gonzales 42)*, Ojojo blanco (*DeWalt et al. 283, Neill & Quevedo 9386, Seidel et al. 5640*), Ojoso (*Vargas C. et al. 5366*), Ojoso blanco (*Del Aguila et al. 95*), Ojoso negro (*Cruz & Terceros 208, Meneces & Terceros 473, D.N. Smith et al. 14160, 14194A, 14215, 14346*), Palo fino (*Cruz & Medina 153*), Periquina hoja menuda (*Meneces & Hartshorn 777*); Tumuqui (Tacana name) (*DeWalt et al. 283*). Brazil: Envira orelha de onça (*Silveira et al. 1094*), Piaçaba (*Campbell et al. 9473a*). Ecuador: Aparina cara caspi (Quichua name) (*Zuleta 364*), Shampipian (*Ortega U. 233*). Peru: Ochabaja (*Begazo 61*), Panjil ruro (*Albán C. 5878, Schunke V. 1203*), Paujil ruro (*Tello 257*), Paujil ruro amarillo (*Kröll 217*).

#### Specimens examined.

**Bolivia.** Beni: Prov. Moxos, 130 km S of San Ignacio, Del *Aguila et al. 95* (U), 27 km from San Borja, along road to Trinidad, 260 m, *D.N. Smith et al. 14160* (U), *14194A* (U), *14215* (MO); Prov. Ballivian, 35 km on the road from Yucumo to Rurrenabaque, 230 m, *D.N. Smith et al. 14346* (MO). La Paz: Prov. Iturralde, Comunidad de Santa Fé, 250 m, *DeWalt et al. 510* (U), Upper Madidi ridge top, 7 km NE of camp, 370–380 m, *Gentry et al. 70691* (MO); Prov. Iturralde, Ixiamas, 90 m, *Couvreur & Vargas 213* (MO, RB); Prov. Sur Yungas, Basin of Río Bopi, Asunta, near Evenay, 690–750 m, *Krukoff 10669* (A, F, G, K, MICH, MO, NY, S, U, UC, US); Prov. Sur Yungas, Upper Río Beni, concession of cooperativa Sapecho, 600 m, *Seidel et al. 5640* (U). Pando: Prov. Manuripi, Batraja, 35 km E of Puerto Rico, 150 m, *Chatrou et al. 453*, *457* (U). Santa Cruz: San Rafael de Amboró, Centro de Estudios Ambientales, 400 m, *Maas et al. 8762* (U); Prov. Ichilo, Parque Nacional Amboró, Campamento Molle, San Germán, road from Yapacaní to Río Ichilo, 400 m, *Maas et al. 8773* (U); Prov. Ichilo, Reserve Forestal `El Choré’, 240 m, *Meneces et al 473, 476* (U); Prov. Ichilo, Parque Nacional Amboró, 23 km S of Buena Vista, along Río Chonta, 420 m, *Nee 36863* (LPB, MO, NY, U); Prov. Ichilo, Parque Nacional Amboró, 4 km SW of Buena Vista, S side of Río Surutu, 315 m, *Nee 39391* (MO, NY, U, WIS); Prov. Ichilo, Reserva Forestal Choré, Río Ibabo, bosque experimental “Elias Meneces”, 180 m, *Neill et al. 9386* (G, MO); Prov. Sara, 400 m, *Steinbach 7248* (A, E, F, G, K, LPB, MO, NY, U); Prov. Ichilo, Parque Nacional Amboró, Río Yapojé and Saguayo, 8 km SW of El Carmén, 360 m, *Vargas C. et al. 1129* (MO), *1133* (NY, MO); Prov. Ichilo, El Carmén, 8 km SSW of Buena Vista, on the way to Huayti, 400 m, *Vargas C. & Mendéz F. 5366* (U). **Brazil.** Acre: Upper Rio Moa near Fazenda Arizona, *Campbell et al. 6196* (U); Fazenda Bom Sossego, between Igarapé Cujubim and Igarapé Jacamin, *Campbell et al. 8633, 9473a* (U); Mun. Cruzeiro do Sul, Igarapé Humaitá, afflente do Rio Juruá, Colocação Várzea Grande, *Cid et el. 10453* U); Mun. Cruzeiro do Sul, Rio Juruá, entrada do Igarapé Viseu, *Cid et al.10535* (U); Mun. Cruzeiro do Sul, Upper Rio Juruá, Fazenda Calila Sara, *Cid et al. 10839* (MO, U), Mun. Sena Madureira, Basin of Rio Purus, Rio Iaco, right bank, Nova Olinda, between Igarapé Santo Antonio and Igarapé Boa Esperança, *Daly et al. 7909* (U); Mun. Marechal Thaumaturgo, Basin of Rio Juruá, Rio Bagé, *Daly et al. 10245* (U); Mun. Manoel Urbano, Rio Chandles (tributary of Rio Purus), downstream from Igarapé Canamarí, *Daly et al. 11234* (U); Colonia Cinco Mil (“Seita do Cipó”), *Nelson 818* (MICH, MO, NY, RB, U, US); Mun. Xapuri, Reserva Extrativista Chico Mendes, Serigal Floresta, Colocação Rio Branco, *Figueiredo 270* (U); Tarauacá, Bacia do Alto Juruá, Rio Tarauacá, Reserva Indígena Praia do Carapanã, Colocação Nova Morada. *Silveira et al. 1094* (U). **Colombia.** Linden s.n. (CGE); Cundinamarca: Mun. Yacopí, vereda El Morro, 405 m, *Rangel et al. 13563* (U). Meta: Parque Nacional Natural Tinigua, Serranía Chamusa, Centro de Investigaciónes Primatológicas La Macarena, *Stevenson 299* (COL). **Ecuador.** Napo: Parque Nacional Yasuní, Pozo petrolero Daimï, 200 m, *Cerón et al. 4227* (MO); Cantón Tena, Estación Biológica Jatun Sacha, 8 km E of Misahuallí, 400 m, *Cerón et al. 5976* (G, GB, MO, U), Cantón Aguarico, Reserva Etnica Huaorani, 260 m, *Dik 1220* (U), Parque Nacional Yasuní, Pozo petrolero Daimï I, Conoco, 230 m, *Hurtado et a1. 21* (MO, U); Cantón Tena, Estación Biológica Jatun Sacha, 8 km E of Misahuallí, 450 m, *Maas et al. 8600* (U), Río Napo, 8 km down the river from Puerto Misahuallí, 400 m, *Neill & Palacios 7130* (U); Reserva Biológica Jatun Sacha, Río Napo, 8 km Río E of Misahualli, 450 m, *Neill & Manning 8014* (U); Estación Experimental INIAP Payamino, 5 km NE of Coca, 250 m, *Palacios et al. 1036* (AAU, F, U); Reserva Biológica Jatun Sacha, Río Napo, 8 km down the river from Misahualli, on right side, 450 m, *Palacios 1421* (U, MO); Cantón Loreto, Payamino-Loreto road, Comunidad Jumandi, 350 m, *Palacios 10950* (F, U); 30 km NNW of Coca, Río Huashito, Site of Proyecto Huashito, 250 m, *T.D. Pennington 10609* (U); Cantón Tena, Estación Biológica Jatun Sacha, 400 m, *Revelo 65* (U); Cantón Orellana, Sector Huashito, 20 km N of Coca, Propriedad de Palmoriente, 250 m, *Rubio 249* (G, GB, K, MO); Estación Biológica Jatun Sacha, confluence of Río Arajuno and Río Puní, 450 m, *Zuleta 39* (U), Estación Biológica Jatun Sacha, 400 m, *Zuleta 364* (U). Pastaza: Cantón Arajuno, Campamentos temporales 11–12, 785 m, *Freire et al. 3468* (U). Sucumbios: Reserva de Producción Faunistica Cuyabeno, 1 km N of Laguna Grande, 265 m, *Valencia et al. 68558* (AAU). **Peru.** Huánuco: Tingo Maria, *Asplund 13193* (G, NY, S); Prov. Puerto Inca, Distr. Yuyapichis, Dantas, 270 m, *Kröll 217* (G, NY), *569* (G), *623* (G), *781* (G); Prov. Pachitea, Distr. Honoria, *Schunke V. 1203* (F, G, GH, K, MO, NY); Distr. Yuyapichis, Prov. Puerto Inca, Unidad Modelo de Manejo y Producción Forestal Dantas, 270 m, *Tello 257* (G, MO, ibidem, *Timaná 143* (G); Shapajilla, 630 m, *Woytkowski 32* (F). Loreto: Prov. Maynas, Yanamono, Explorama Tourist Camp, Río Amazonas halfway between Indiana and mouth of Río Napo, 130 m, *Gentry et al. 42403* (MO). Madre de Dios: Prov. Tambopata, Río Heath, Pto. San Antonio, 210 m, *Aguilar & Castro 916* (U), *1076* (U), Communidad Nativa, Sonene, ca 8 km above confluence with Río Madre de Dios, 180 m, *Alexiades et al. 1210* (U); Prov. Manú, Parque Nacional de Manu, Estación Biológico Cocha Cashu, *Chatrou et al. 157* (U), Parque Nacional “Bahuaja-Sonene”, *Díaz et al. 9037* (U), *Díaz & Ramírez 9548* (U); Cashucocha Camp, Río Manu, Parque Nacional de Manu, 380 m, *Gentry et al. 26821* (U), *26952* (F); Cocha Cashu Biológical Station, Parque Nacional de Manu, 400 m, *Gentry 43334* (U), *Gentry 43537* (MO); Tambopata Nature Reserve, junction of Río La Torre and Río Tambopata, 250 m, *Gentry et al. 57751* (MO); Prov. Tambopata, 15 km ENE of Puerto Maldonado, Cusco Amazónico, 200 m, *Gentry et al. 68713* (MO); Prov. Manu, Puerto Maldonado, Los Amigos Biological Station, Madre de Dios River, *Janovec et al. 2014* (U), ibidem, 270 m, *Maceda 7* (U); Prov. Tambopata, Cusco Amazónico, 200 m, *Núnez et al. 10607* (MO); Prov. Manu, Río Manu, Pakitsa - behind station, 350 m, *Sobrevila et al. 1782* (F); Prov. Tambopata, Las Piedras, Cusco Amazónico, 200 m, *Timaná et al. 1722, 2795, 3060* (MO). San Martín: Prov. Huallaga, Barranca, 380 m, *Albán C. 5878* (F); Juanjuí, Alto Río Huallaga, 400 m, *Klug 3798* (F, GH, K, MO, NY, S); Moyobamba - Tarapoto, km 20, 1000 m, *T.D. Pennington & Daza 16708* (U; Prov. Mariscal Caceres, Distr. Tocache Nuevo, Quebrada de Saule Grande (margen derecho del Río Huallaga), *Schunke V. 4387* (G, GH, K, MO, NY, US). Ucayali: Prov. Pucallpa, Estación Experimental Alexander von Humboldt, *Begazo 61*, *99* (U); Prov. Coronel Portillo, Humboldt station, near Campo Verde, ca. 34 km SW of Pucallpa, 200 m, *Morawetz & Wallnöfer 11-19985* (U); Prov. Coronel Portillo, Bosque Nacional Alexander von Humboldt, km 68 of Pucallpa-Tingo Maria road, 270 m, *Oliveira 15* (MO); Prov. Ucayali, Distr. Pampa Hermosa, Pampa Hermosa, 240 m, *Reynel R. 832* (U); Prov. Coronel Portillo, Bosque Nacional Alexander von Humboldt, km 68 of Pucallpa-Tingo Maria road, 270 m, *Salazar 32* (MO); Canchahuayo, Río Ucayali, 500 m, *Vásquez et al. 6958* (AAU, F, NY, MO, U).

#### Notes.

This species is easily recognizable by a combination of oval and on both sides rounded leaves with an olive green colour, long and narrow petals (often drying blackish), and stipes and apicules that are excentrically placed.

*Maas et al. 8773* from Bolivia: Inner layer of monocarps ca. 5 mm thick, orange-yellow, sweet and edible.

### Pseudephedranthus

Taxon classificationPlantaeMagnolialesAnnonaceae

Aristeg.

Pseudephedranthus Aristeg., Memoirs of the New York Botanical Garden 18(2): 43. f. 10. 1969.

#### Type.

*Pseudephedranthus fragrans* (R.E.Fr.) Aristeg. (= *Ephedranthus fragrans* R.E.Fr.).

#### Description.

*Trees*; young twigs glabrous. *Leaves*: distichous, simple, entire, shortly petiolate, exstipulate; lamina medium-sized, elliptic, venation brochidodromous, primary vein raised above. *Inflorescences* axillary, 1–4-flowered with 2^nd^ order flowers originating from axils of lower bracts (or possibly also through accessory buds), often persisting on older leafless branchlets, pedicels with articulation in lower part and with 3–5 bracts, the uppermost bract above the articulation. *Flower*s actinomorphic, bisexual or staminate (androdioecious), 3-merous, perianth consisting of one whorl of sepals and two whorls of petals; sepals 3, valvate, basally connate, much shorter than the petals; petals 6, imbricate, elliptic, free, subequal; staminate flowers: torus conical, stamens numerous, extrorse, filament very short, apical prolongation of connective broadly ovoid in basal stamens to discoid in distal stamens; bisexual flowers: torus slightly raised, stamens numerous, but less so than in staminate flowers, apical prolongation of connective broadly ovoid; carpels numerous, spirally arranged, free, ovary 1-locular, with 1 basal ovule, style absent, stigma ovoid, papillate. *Fruit* apocarpous, composed of few, indehiscent monocarps, these ellipsoid, distinctly stipitate. *Seed* 1, pale brown, rumination lamellate in 2–4 parts, raphe a distinct groove.

#### Distribution.

Two species in the Amazon regions of Venezuela and Brazil, and in Guyana and Suriname.

#### Key to the species of *Pseudephedranthus*

**Table:** 

1a	Petioles 3–5 mm long; leaf base acute to attenuate; outer side of outer petals (rather) densely covered with appressed hairs, inner side of outer petals and both sides of inner petals densely covered with white or greyish white curly hairs (Suriname, and the Brazilian state of Pará) ***P. enigmaticus***
1b	Petioles 8–12 mm long; leaf base obtuse to less often acute, the extreme base shortly atttenuate or not; outer side of outer and inner petals densely covered with appressed, not curly hairs to ca. 0.1 mm, inner side of outer and inner petals sparsely covered with appressed hairs or glabrous (Upper Río Negro region of Brazil and Venezuela)	***P. fragrans***

### Pseudephedranthus enigmaticus

Taxon classificationPlantaeMagnolialesAnnonaceae

Maas & Westra, sp. nov .

urn:lsid:ipni.org:names:77165709-1

Figs 4 , 5

#### Diagnosis.

Differing from *P. fragrans* by shorter petioles. Moreover, petals in *P. enigmaticus* are for a large part covered by a very dense indument of curly hairs, those of *P. fragrans* covered by a less dense indument of appressed hairs. Also, seeds in *P. enigmaticus* are ellipsoid instead of ovoid and smaller than in *P. fragrans*.

#### Type.

SURINAME, Sipaliwini, Central Suriname Nature Reserve, ca. 4 km ENE of Kayserberg Airstrip, alt. 235 m, 4 June 2003, *Evans et al. 3437* (holotype WAG! [barcode WAG.1584983]; isotype L! [barcode L.3724851]).

#### Description.

*Tree*, 3–15 m tall, 12–20 cm diam.; young twigs glabrous. *Leaves*: petioles 3–5 by 1–2 mm; lamina narrowly elliptic, 12–22(–26) by 4–6(–9) cm (index 2.8–4), chartaceous, pale gray to greenish gray above *in sicco*, somewhat bullate above *in vivo*, greenish brown to pale brown below *in sicco*, base acute, apex acuminate (acumen 5–10 mm long), primary vein raised above, secondary veins 6–10 on either side of primary vein, raised above, smallest distance between secondary veins and margin 4–7 mm, tertiary veins raised, rarely flat above, reticulate. Only staminate flowers seen, *Inflorescence* axillary, 1–2(–several)-flowered, pedicels 3–12 mm by 0.5–2 mm, rather densely to sparsely covered with erect to appressed, brown hairs to ca. 1 mm long, soon glabrous; bracts 4–5, depressed ovate, 1–2 mm long, outer side rather densely to sparsely covered with erect to appressed, brown hairs; flower buds ellipsoid; sepals shallowly ovate-triangular, ca. 2 by 2–3 mm, outer side rather densely to sparsely covered with erect to appressed, brown hairs; petals white, tinged with pale green *in vivo*, oblong-elliptic to narrowly so, 7–12 by 3–6 mm, outer side of outer petals densely to rather densely covered with appressed, brown hairs, inner side densely covered with whitish or greyish-white, curly hairs except for the glabrous base, outer side and apical part of inner petals densely covered with curly, white hairs; staminate torus conical, 2–2.5 mm long, ca. 1 mm diam. at base; stamens ca. 50, 2–2.5 mm long, apical prolongation of connective discoid, broadly elliptic. *Monocarps* 3–15, green *in vivo*, black *in sicco*, ellipsoid, 12–32 by 7–15 mm, glabrous or sparsely covered with appressed hairs, apex rounded, wall 0.2–0.5 mm thick, stipes 1–4 mm long, 1–1.5 mm diam. *Seed* ellipsoid, 12–19 by 7–10 mm, pale brown, transversely striate.

#### Distribution.

Guyana, Suriname, and the Brazilian state of Pará. Fig. 1.

#### Habitat and ecology.

In periodically inundated or non-inundated forest, on sandy or loamy soil, alt. 100–600 m. Flowering: May, June; fruiting: June, July, September.

#### Specimens examined.

**Brazil**. Pará: Parque Indígena do Tumucumaque, Rio Parú de Oeste, Missão Tiriyo, *Cavalcante 2579* (U); Rio Maicuru, Igarapé do Mutum, 31/2 hrs. por canoa de motor de poupa acima da pista de pouso do Lageiro, *Jangoux & Ribeiro 1555* (L, RB); W bank of Rio Maicuru, ca. 23 km upstream from Lageira airstrip, N side of Mutum stream, *Strudwick et al. 3808* (U). **Guyana**. Takutu-U Region, Rupununi River, between Kwattamang Landing and Rewa Village, 100 m, *Clarke et al. 6750* (NY, US). **Suriname**. Suriname, Sipaliwini District, Sipaliwini River, Werehpai, 5 September 2010, *Bánki et al. 1674* (L); Sipaliwini District, Sipaliwini River, *Bánki et al. 1579* (L); Sipaliwini, vicinity of camp on W bank of Zuid River, across river (i.e. W and outside of) Central Suriname Nature Reserve, ca. 10 km straight-line distance SSE of Kayserberg Airstrip, 240 m, *Evans et al. 3485* (L); Sipaliwini, Central Suriname Nature Reserve, on S slope of the first peak in Eilerts de Haan mountain range, ca. 7 km ENE of Kayserberg Airstrip, 400–600 m, *Herrera C. et al. 9959* (L, WAG); Distr. Nickerie, area of Kabalebo Dam project, 30–130 m, *Lindeman & de Roon 752* (U); Sipaliwini, Morro Grande camp forest island, 6 km W of Morro Grande dome, 360 m, *Oldenburger et al. 416* (U); Sipaliwini, Central Suriname Nature Reserve, 2–5 km SE of E end of Kayserberg Airstrip, 235 m, *Rosário 1796* (L); Sipaliwini, Central Suriname Nature Reserve, 2–5 km ENE of Kayserberg Airstrip, 235 m, *Rosário et al. 1829* (MO); Sipaliwini, Central Suriname Nature Reserve, vicinity of camp at southern base of the first peak in Eilerts de Haan mountain range, 250–350 m, *C.S. & D.O. Rosário 2176* (L).

#### Notes.

Material of this species had previously been filed in herbaria under different generic names such as *Cremastosperma*, *Guatteria*, *Klarobelia*, *Malmea*, *Oxandra*, and *Rollinia* (which is quite aberrant!). The confusion is aptly expressed in the epithet “enigmaticus”. This new species fits quite well, however, within the genus *Pseudephedranthus* (segregated from *Ephedranthus* by Aristeguieta in 1969), among others by the leaf venation, fruit and seed structure, and the strong similarity of the flowers. We acknowledge the fact that Pirie et al. (2006) demonstrated that *Pseudephedranthus fragrans* is nested in *Klarobelia*. From a morphological point of view (leaves and venation; flower morphology) this is quite surprising, given that overall morphology of *Klarobelia* is homogenous, and *Pseudephedranthus* is deviant from the general *Klarobelia* morphology. Therefore, we prefer to describe this new species in *Pseudephedranthus* to reflect the morphological similarity to *P. fragrans*. *P. enigmaticus* is distinct from *P. fragrans* by shorter petioles and, particularly, by the much denser indument of small curly hairs on most of the inner side of the petals (versus mostly small straight hairs). Also, seeds in *P. enigmaticus* are ellipsoid and 12–19 by 7–10 mm in contrast to *P. fragrans* were they are ovoid and larger (25–30 by 13–15 mm).

The specimens investigated here were either fruiting or flowering, the flowers all being staminate. Carpel bearing flowers are still needed to complete the description.

### Pseudephedranthus fragrans

Taxon classificationPlantaeMagnolialesAnnonaceae

(R.E.Fr.) Aristeg.

Figs 6 , 7

Pseudephedranthus fragrans (R.E.Fr.) Aristeg., Memoirs of the New York Botanical Garden 18(2): 43. f. 10. 1969.
Ephedranthus fragrans R.E.Fr., Memoirs of the New York Botanical Garden 9(3): 327. 1957.

#### Type.

VENEZUELA. Amazonas: Río Negro, occasional on lower slopes of Piedra Nunca, just N of Piedra de Cucuy, 100–150 m, 10 April 1953, *Maguire & Wurdack 34954* (holotype S [barcode S.85211]; isotypes F, GH, NY, P, S).

#### Description.

*Tree*, 10–20 m tall, 15–30 cm diam.; young twigs glabrous, fissured, covered with a white, waxy surface. *Leaves*: petioles 8–12 by 1–2 mm; lamina lamina elliptic to narrowly elliptic, (5–)10–25 by (2.5–)4–7(–9) cm (index 2–3), chartaceous to coriaceous, dark greenish-brown above, pale green below, shiny and glabrous on both sides, base obtuse to less often acute, the extreme base shortly attenuate or not, apex shortly acuminate (acumen to ca. 15 mm long), secondary veins 5–9 on either side of primary vein, raised above, smallest distance between secondary veins and margin 2–6 mm, tertiary veins raised above, reticulate. *Inflorescence* axillary, 1–2(–more)-flowered, usually on older parts of branchlets; pedicels 4–8 by ca. 1 mm, fruiting pedicels to ca. 10 by 2–4 mm, densely covered with appressed hairs to ca. 0.1 mm long; bracts 3–5, triangular, 0.7–1 mm long, outer side densely covered with appressed hairs to ca. 0.1 mm long; flower buds broadly ovoid; sepals shallowly ovate-triangular, 1.5–2 by 3 mm, outer side densely covered with appressed hairs to ca. 0.1 mm long; petals white to cream *in vivo*, narrowly elliptic, 9–15 by 3–5 mm, outer side densely covered with appressed hairs to ca. 0.1 mm long; staminate flowers: torus concave, ca. 2.5 mm long, stamens ca. 50, 1.5–2 mm long, basal stamens with a broadly ovoid prolongation of connective, the distal ones with a discoid prolongation of connective, carpels absent; bisexual flowers: torus slightly raised, ca. 1 mm long, stamens ca. 15, 1.5–2.5 mm long, apical prolongation of connective broadly ovoid, carpels ca. 25, ca. 3 mm long, densely covered with appressed hairs to ca. 0.1 mm long, stigma ca. 1 mm long. *Monocarps* 3–10, green, maturing orange *in vivo*, pale brown *in sicco*, ellipsoid, 25–32 by 12–15 mm, apex rounded, sparsely covered with appressed hairs to ca. 0.4 mm long to glabrous, wall 0.2{–1} mm thick; stipes 2–4 by 1.5 mm. *Seed* ovoid, 25–30 by 13–15 mm, pale brown, transversely striate.

#### Distribution.

Restricted to the Upper Rio Negro region of Brazil and adjacent Venezuela. Fig. 1.

#### Habitat and ecology.

In lowland rain forest (in forested hills at the base of Piedra de Cucuy, Maas, pers. comm.). At elevations of 50–600 m. Flowering: April, December; fruiting: October, December.

#### Specimens examined.

**Venezuela**. Amazonas: Río Negro, forested base of Piedra de Cucuy, 100–200 m, *Maas et al. 6878* (INPA, MO, NY, RB, S, U, WU). **Brazil**. Amazonas: Rio Cauaburí, Caño Tucano, 125 m, *Maguire et al. 60189* (F, GH, MO, NY, S), *60191* (MO, NY, S, US); Rio Negro, Rio Cauaburí, Rio Tucano, Camp Tucano (Camp No. 2), 850 ft., *Maguire et al. 60332* (MO); between Palmito Camp and Camp Tatu, 400–600 m, *N.T. Silva & Brazão 60683* (NY, US); Rio Negro, Rio Cauaburí, Rio Maturacá, between Camp III and Maloca, *N.T. Silva & Brazão 60738* (NY).

**Figure 6.**
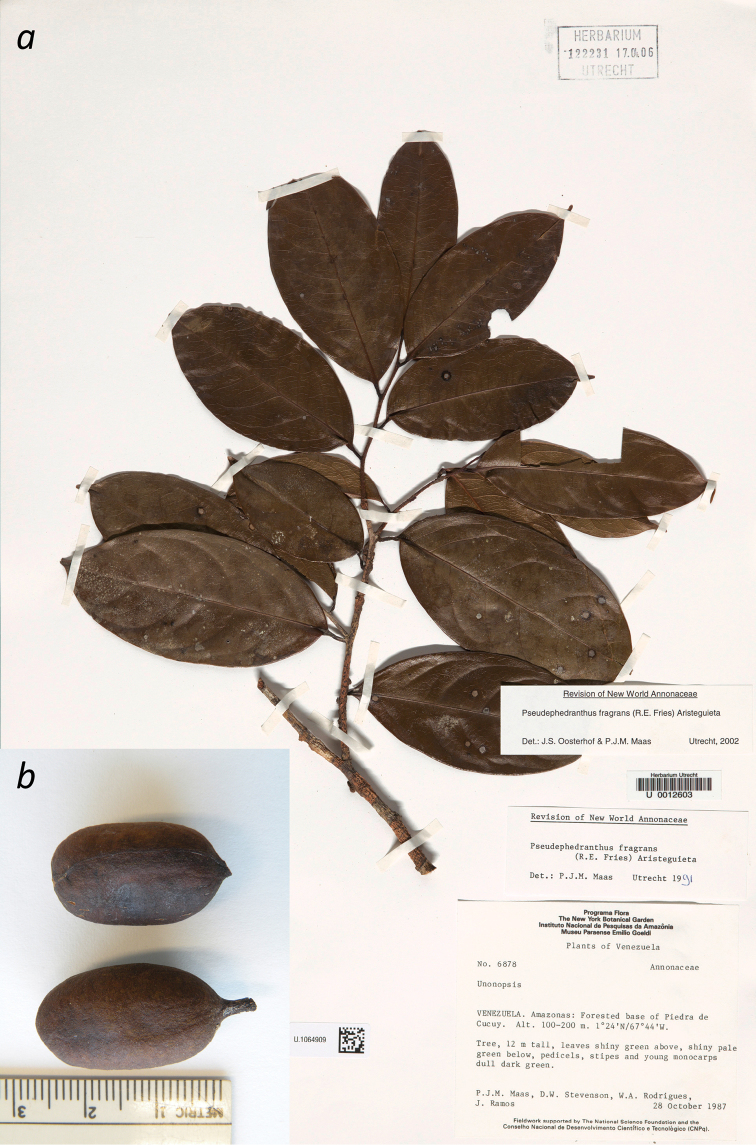
**a** Branch of *Pseudephedranthus fragrans* **b** Monocarps (*Maas et al. 6878*, U).

**Figure 7.**
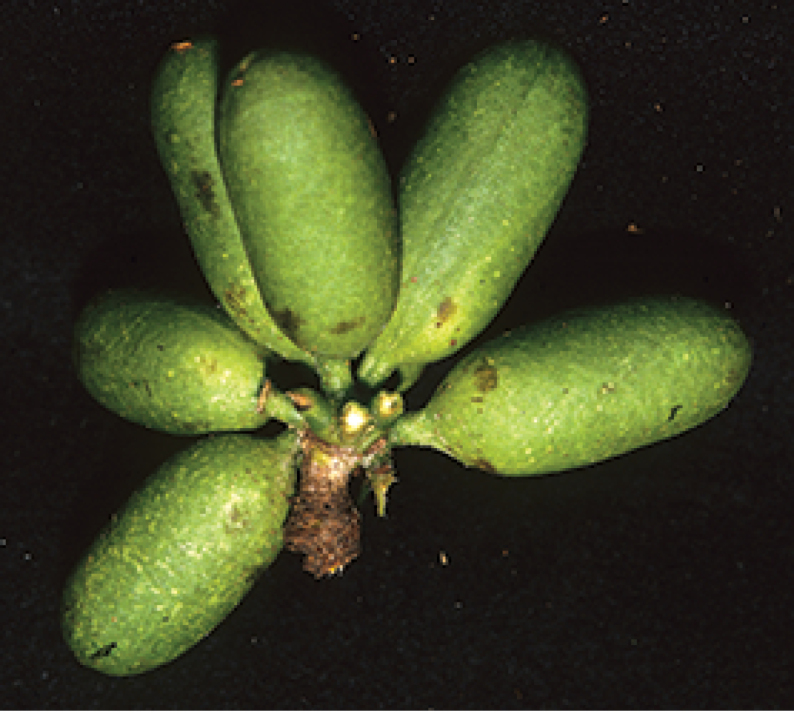
Fruit of *Pseudephedranthus fragrans*. Venezuela, Amazonas: Piedra de Cucuy (*Maas et al. 6878*).

## Supplementary Material

XML Treatment for Ruizodendron

XML Treatment for Ruizodendron ovale

XML Treatment for Pseudephedranthus

XML Treatment for Pseudephedranthus enigmaticus

XML Treatment for Pseudephedranthus fragrans
